# Fabrication of a hierarchical NiTe@NiFe-LDH core-shell array for high-efficiency alkaline seawater oxidation

**DOI:** 10.1016/j.isci.2023.108736

**Published:** 2023-12-15

**Authors:** Xuexuan Ju, Xun He, Yuntong Sun, Zhengwei Cai, Shengjun Sun, Yongchao Yao, Zixiao Li, Jun Li, Yan Wang, Yuchun Ren, Binwu Ying, Yongsong Luo, Dongdong Zheng, Qian Liu, Lisi Xie, Tingshuai Li, Xuping Sun, Bo Tang

**Affiliations:** 1 School of Materials and Energy, University of Electronic Science and Technology of China, Chengdu, Sichuan 611731, China; 2 Institute of Fundamental and Frontier Sciences, University of Electronic Science and Technology of China, Chengdu, Sichuan 610054, China; 3 College of Chemistry, Chemical Engineering and Materials Science, Shandong Normal University, Jinan, Shandong 250014, China; 4 Institute for Advanced Study, Chengdu University, Chengdu, Sichuan 610106, China; 5 Laoshan Laboratory, Qingdao, Shandong 266237, China

**Keywords:** Materials chemistry, Energy materials

## Abstract

Herein, a hierarchical NiTe@NiFe-LDH core-shell array on Ni foam (NiTe@NiFe-LDH/NF) demonstrates its effectiveness for oxygen evolution reaction (OER) in alkaline seawater electrolyte. This NiTe@NiFe-LDH/NF array showcases remarkably low overpotentials of 277 mV and 359 mV for achieving current densities of 100 and 500 mA cm^−2^, respectively. Also, it shows a low Tafel slope of 68.66 mV dec^−1^. Notably, the electrocatalyst maintains robust stability over continuous electrolysis for at least 50 h at 100 mA cm^−2^. The remarkable performance and hierarchical structure advantages of NiTe@NiFe-LDH/NF offer innovative insights for designing efficient seawater oxidation electrocatalysts.

## Highlights

• A hierarchical NiTe@NiFe-LDH/NF acts as a superb electrocatalyst for seawater oxidation
• It demands low overpotentials of 277 mV for achieving a current density of 100 mA cm^−2^
• It maintains robust stability over continuous electrolysis for 50 h at 100 mA cm^−2^

## Graphical abstract

**Figure:**
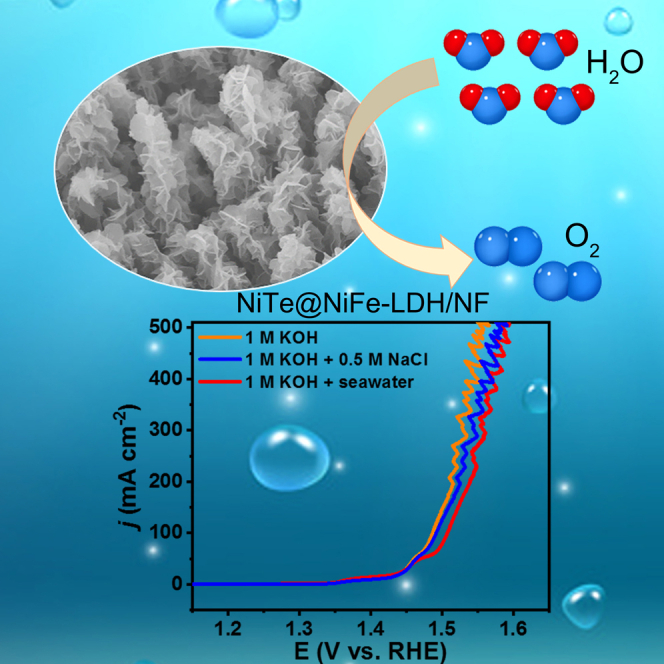

## Introduction

Hydrogen, a high energy density green energy source, with water as its sole combustion product, offers a compelling alternative to fossil fuels.^1^^,^^2^ Among the current hydrogen generation techniques, water electrolysis stands out for its single-step hydrogen generation and environmentally friendly attributes.^3^^,^^4^^,^^5^^,^^6^^,^^7^ Its progress, nevertheless, is constrained by the limited availability of freshwater resources on Earth. This limitation has spurred interest in seawater electrolysis as a potential avenue for hydrogen production, leveraging the abundance of seawater resources.^8^^,^^9^^,^^10^^,^^11^^,^^12^^,^^13^ Yet, the efficiency of seawater electrolysis encounters a challenge. Abundant chlorine ions (Cl^−^) in seawater tend to outcompete the oxygen evolution reaction (OER) at the anode for the kinetically swift two-electron chlorine evolution reaction (CER). This not only interferes with the OER but also corrodes anode catalysts, generating hypochlorite.^14^^,^^15^^,^^16^^,^^17^ Significantly, in alkaline electrolytes (e.g., 1 M KOH + seawater), the thermodynamic potential difference between the OER and CER can exceed 480 mV.^18^^,^^19^ To mitigate Cl^−^ interference, it becomes plausible to achieve high current densities within a thermodynamic potential range of ≤480 mV.

Non-noble metal-based catalysts have demonstrated significant potential in seawater oxidation.^20^^,^^21^^,^^22^^,^^23^^,^^24^^,^^25^ Among these, nickel-iron (NiFe) based catalysts, particularly those with two-dimensional nanosheet structures such as NiFe-layered double hydroxide (LDH), have garnered widespread attention for their abundance, facile synthesis, and high OER activity in alkaline seawater.^26^^,^^27^^,^^28^ The poor electrical conductivity of NiFe-LDH remains a limitation, which leads to inefficient electron-ion transport at its interface.^28^ Various strategies such as heteroatom doping,^29^^,^^30^ anion exchange,^31^^,^^32^ hybridizations with carbon,^33^^,^^34^ etc., have been proposed to enhance the catalytic performance of NiFe-LDH. Constructing a 3D hierarchical structure using a conductive core is a promising strategy for improving its electrical conductivity and exposing more active sites, thus promoting catalytic activity.^35^^,^^36^^,^^37^^,^^38^^,^^39^ Wang et al. reported the fabrication of NiFe-LDH on NiMoO_4_ nanorod, increasing the active surface area, allowing for faster kinetics.^35^ Our research group further introduced NiFe-LDH on NiMoS_x_ microcolumn, which exhibited superior OER activity.^39^ Tellurium, as a chalcogenide, is more metallic and thus more conductive than its fellow oxygen and sulfur.^40^^,^^41^ Nickel telluride (NiTe) has been demonstrated as fast electron-mass transport, facilitated by a pronounced covalent interaction within the Ni-Te bond.^40^^,^^41^^,^^42^^,^^43^ The assembly of a hierarchical structure comprising NiFe-LDH shell and NiTe core is anticipated to be an ideal seawater oxidation electrocatalyst, a proposition that remains unprecedented.

In this work, we present the fabrication of well-dispersed NiFe-LDH nanosheet on NiTe nanorod array supported on Ni Foam (NiTe@NiFe-LDH/NF) as a highly active seawater oxidation electrocatalyst. To drive a current density (*j*) of 100 mA cm^−2^, such NiTe@NiFe-LDH/NF requires an overpotential of 277 mV in alkaline seawater. Furthermore, the catalyst also demonstrates remarkable electrochemical stability, maintaining its performance for a continuous duration of 50 h without any observable deterioration.

## Results and discussion

**Figure 1:**
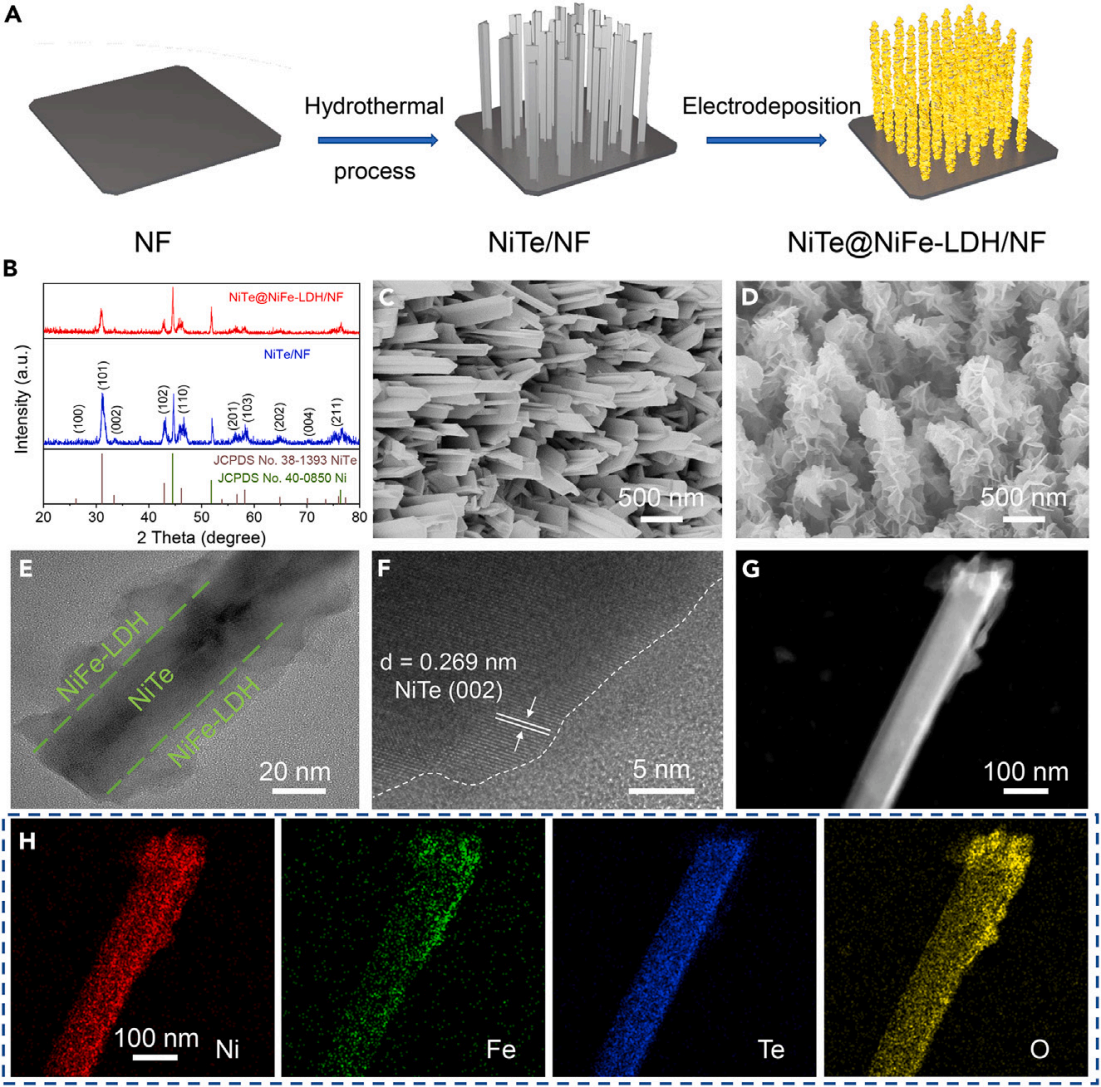
Structural characteristics of NiTe@NiFe-LDH/NF (A) Schematic illustration of the formation of NiTe@NiFe-LDH/NF. (B) XRD patterns of NiTe@NiFe-LDH/NF and NiTe/NF. (C) SEM image of NiTe/NF. (D) SEM image of NiTe@NiFe-LDH/NF. (E) TEM image and (F) HRTEM image of NiTe@NiFe-LDH. (G) HAADF-STEM image and (H) EDS elemental mapping images of NiTe@NiFe-LDH.

Figure 1A shows a two-step synthesis of NiTe@NiFe-LDH/NF. Firstly, NiTe nanorods were grown on NF (NiTe/NF) by a hydrothermal method. NiFe-LDH nanosheets were then electrodeposited on NiTe/NF to get NiTe@NiFe-LDH/NF. In Figure 1B, the X-ray diffraction (XRD) pattern of NiTe/NF indicates the formation of NiTe nanorods on NF (JCPDS No. 38–1393),^41^ three distinct peaks at 44.4°, 52.4°, and 76.5° correspond to characteristic diffraction peaks of metallic Ni within the NF substrate (JCPDS No. 40–0850). No discernible alterations are observed after the electrodeposition process, which is consistent with the inherent amorphous nature of the deposited NiFe-LDH.^44^ The morphology of NiTe nanorods is displayed T-shape in Figure 1C, while in Figure 1D, NiFe-LDH nanosheets are uniformly deposited on the surface of NiTe nanorods. For comparison, the morphology of NiFe-LDH/NF, displayed in Figure S1, exhibits that nanosheets are evenly distributed on NF. Transmission electron microscopy (TEM) image reveals a distinct core-shell structure of NiTe@NiFe-LDH, with clearly discernible boundaries between NiTe and NiFe-LDH, as indicated by green dashed lines in Figure 1E. In Figures 1F and 1A high-resolution TEM (HRTEM) image displays a crystal lattice distance of 0.269 nm, corresponding to the (002) plane of NiTe, and a clear demarcation between crystalline NiTe and amorphous NiFe-LDH. High-angle annular dark-field scanning TEM (HAADF-STEM) image (Figure 1G) further confirms the core-shell configuration of NiTe@NiFe-LDH. Energy dispersive spectrometer (EDS) elemental mapping images in Figure 1H exhibit the even distribution of Ni, Fe, Te, and O elements for NiTe@NiFe-LDH. This elemental distribution provides conclusive evidence that NiTe nanorod is uniformly enveloped by NiFe-LDH.

**Figure 2:**
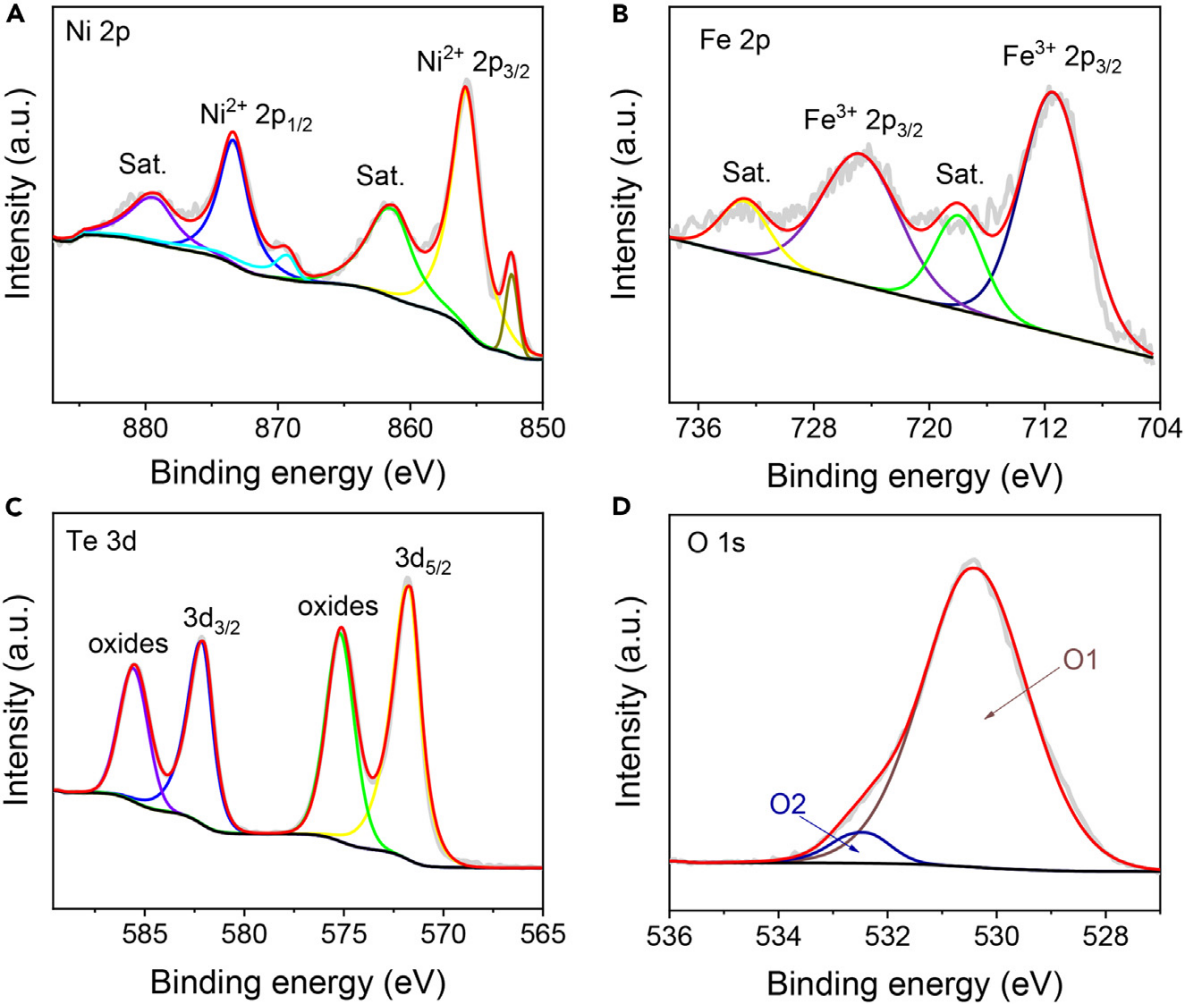
XPS spectra of NiTe@NiFe-LDH/NF (A) XPS spectra in the Ni 2p region. (B) XPS spectra in the Fe 2p region. (C) XPS spectra in the Te 3d region. (D) XPS spectra in the O 1s region.

**Figure 3:**
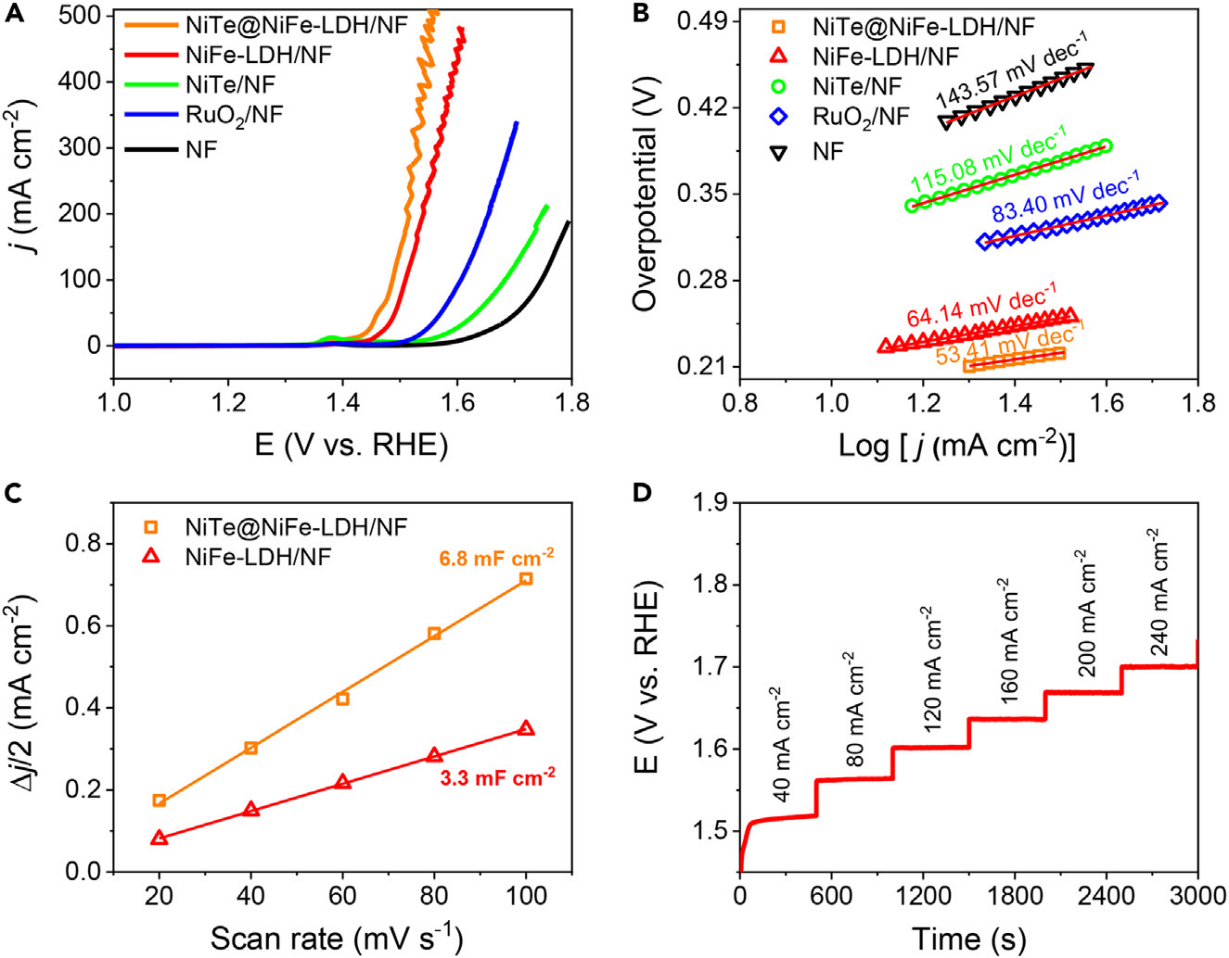
Electrochemical tests in 1 M KOH (A) LSV curves and (B) corresponding Tafel plots of NiTe@NiFe-LDH/NF, NiFe-LDH/NF, NiTe/NF, NF, and RuO_2_/NF. (C) *C*_dl_ plots of NiTe@NiFe-LDH/NF and NiFe-LDH/NF. (D) Multistep chronopotentiometric test of NiTe@NiFe-LDH/NF without iR correction.

The surface chemical state of the NiTe@NiFe-LDH/NF can be elucidated through X-ray photoelectron spectroscopy (XPS). The Ni 2p spectrum of NiTe@NiFe-LDH/NF (Figure 2A) reveals two discernible peaks at 855.7 and 873.3 eV, corresponding to Ni 2p_3/2_ and Ni 2p_1/2_ of Ni^2+^. The dual peaks located at 861.3 and 879.2 eV are attributed to satellite peaks (Sat.). Besides, additional peaks at 852.4 and 869.5 eV align with Ni^0^ of NF.^45^^,^^46^^,^^47^ The Fe 2p spectrum (Figure 2B) illustrates two prominent peaks at 711.5 eV for Fe^3+^ 2p_3/2_ and 725.0 eV for Fe^3+^ 2p_1/2_. The additional two peaks at 718.1 and 733.0 eV correspond to Sat.^47^^,^^48^^,^^49^ Regarding the Te 3d spectrum shown in Figure 2C, two peaks at 572.7 and 583.1 eV can be attributed to Te^2−^ 3d _5/2_ and Te^2−^ 3d _3/2_ of Te^2−^ in NiTe, respectively. The presence of two additional Te 3d peaks with binding energies of 576.1 and 586.5 eV suggests a potential association with the surface oxidation of Te.^40^ The O 1s region indicates two distinguishable peaks, labeled as O1 and O2, situated at 530.4 and 532.5 eV, respectively. These peaks correspond to metal-OH (M-OH) and surface-adsorbed oxygen species (Figure 2D).^50^^,^^51^

the electrochemical performance of NiTe@NiFe-LDH/NF for OER was initially assessed in a 1 M KOH electrolyte. Based on the Linear Sweep Voltammetry (LSV) curves obtained for different electrodeposition times (as illustrated in Figure S2), it was determined that a 60-s electrodeposition time provided the most favorable conditions for OER. LSV curves for NiTe@NiFe-LDH/NF, NiFe-LDH/NF, NiTe/NF, NF, and commercially available RuO_2_ loaded on NF (RuO_2_/NF) were presented in Figure 3A. Remarkably, NiTe@NiFe-LDH/NF exhibited significantly enhanced electrocatalytic activity compared to the other four electrode configurations. At a *j* of 100 mA cm^−2^, NiTe@NiFe-LDH/NF demonstrated a particularly low overpotential of 257 mV. This overpotential was substantially lower than those for NiFe-LDH/NF (280 mV), NiTe/NF (451 mV), benchmark RuO_2_/NF (370 mV), and NF (517 mV). In Figure 3B, the Tafel slope value of NiTe@NiFe-LDH/NF was found to be the lowest at 53.41 mV dec^−1^ compared to NiFe-LDH/NF (64.14 mV dec^−1^), NiTe/NF (115.08 mV dec^−1^), RuO_2_/NF (83.40 mV dec^−1^), and NF (143.57 mV dec^−1^). This suggests that NiTe@NiFe-LDH/NF exhibits faster kinetics during the OER process. Electrochemical impedance spectroscopy then revealed a smaller charge transfer resistance for NiTe@NiFe-LDH/NF (1.37Ω) compared to that of NiFe-LDH/NF (7.74Ω) (Figure S3), indicating improved charge transfer and reaction kinetics for NiTe@NiFe-LDH/NF. As depicted in Figures 3C and S4, NiTe@NiFe-LDH/NF possessed a double-layer capacitance (*C*_dl_) of 6.8 mF cm^−2^ in 1 M KOH, nearly double that of NiFe-LDH/NF (3.3 mF cm^−2^). Consequently, the increased electrochemical active surface area and active sites of NiTe@NiFe-LDH/NF contribute to its enhanced OER performance. The integration of a conductive NiTe core with NiFe-LDH not only improves conductivity and reaction kinetics but also introduces more active sites, thus enhancing the OER activity of the anode catalyst. Moreover, a multi-step chronopotentiometric curve (from 40 mA cm^−2^ to 240 mA cm^−2^) was presented in Figure 3D, suggesting efficient mass transportation, excellent mechanical durability, and conductivity of the catalyst.

**Figure 4:**
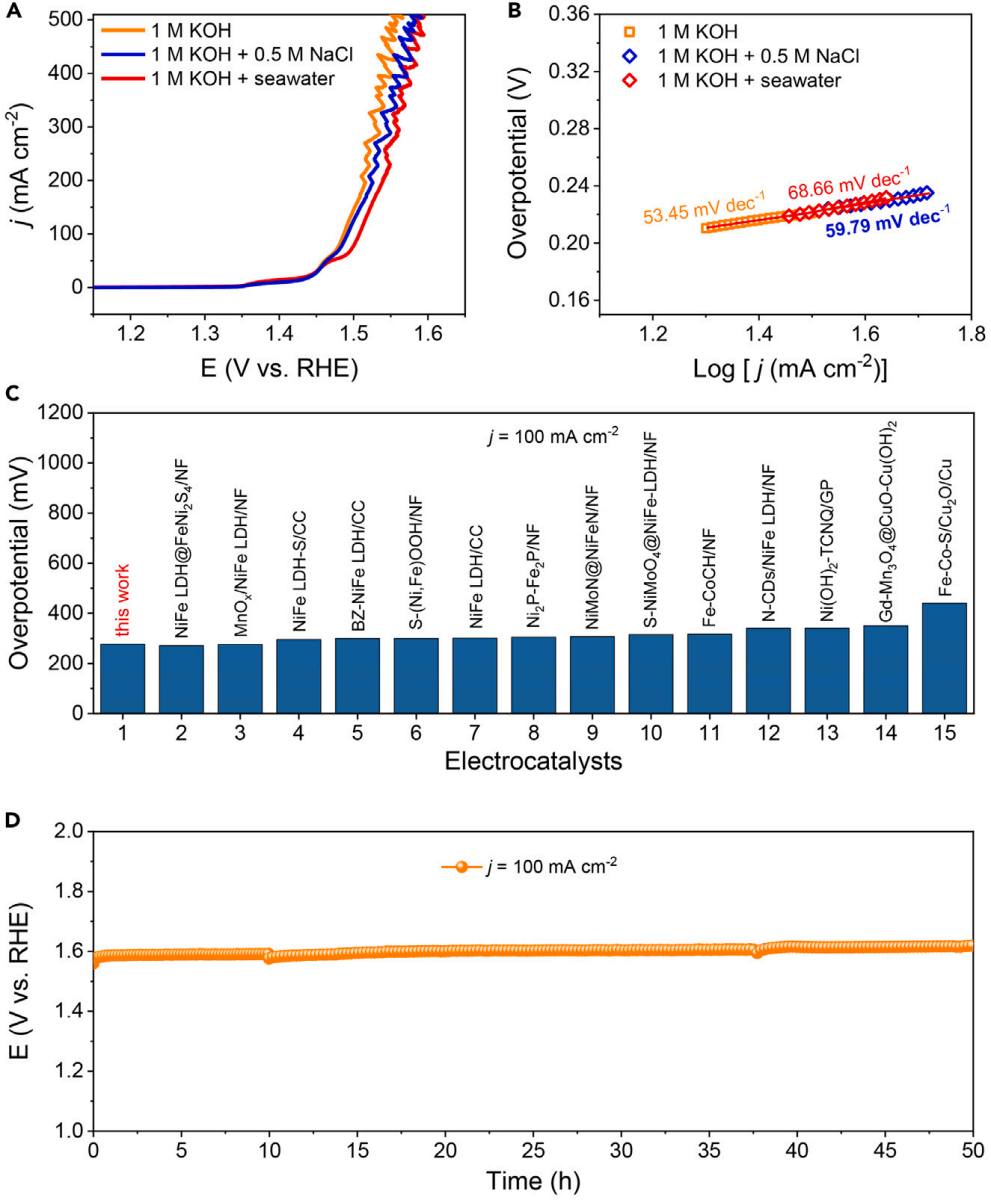
Electrochemical tests in alkaline seawater (A) LSV curves and (B) corresponding Tafel plots of NiTe@NiFe-LDH/NF in 1 M KOH, alkaline stimulated seawater, and alkaline seawater. (C) Comparison of overpotentials required to obtain the *j* of 100 mA cm^−2^ between NiTe@NiFe-LDH/NF and previously reported self-supported catalysts in alkaline seawater. (D) Chronopotentiometry test of NiTe@NiFe-LDH/NF at the *j* of 100 mA cm^−2^ in alkaline seawater (without iR correction).

Following the evaluation of NiTe@NiFe-LDH/NF’s OER catalytic activity in 1 M KOH, its performance was tested as an anode for OER in alkaline simulated seawater (1 M KOH +0.5 M NaCl) and alkaline seawater (1 M KOH + seawater). Illustrated in Figure 4A, the LSV curves from the two mentioned electrolytes closely resembled the curve in 1 M KOH. Notably, the overpotential in alkaline seawater exhibited a mere 20 mV increase compared to that in 1 M KOH. The OER catalytic kinetics of NiTe@NiFe-LDH/NF were further assessed by Tafel slopes in alkaline simulated seawater (59.79 mV dec^−1^) and alkaline seawater (68.66 mV dec^−1^), slightly surpassing that in 1 M KOH (Figure 4B). This trend suggests the swift OER catalytic kinetics of NiTe@NiFe-LDH/NF. Remarkably, the overpotentials required to achieve the *j* of 100, 200, and 500 mA cm^−2^ for NiTe@NiFe-LDH/NF were 277, 309, and 359 mV, respectively (Figure S5). These values surpass those of many reported self-supported electrocatalysts for OER in alkaline seawater (Figure 4C; Table S1). To assess the stability of NiTe@NiFe-LDH/NF in alkaline seawater, an LSV curve was obtained after 1000 CV cycles (Figure S6). No discernible current loss was observed compared to the initial curve before cycling. Furthermore, in Figure 4D, the stability of NiTe@NiFe-LDH/NF was assessed by chronopotentiometry at a *j* of 100 mA cm^−2^ over a span of 50 h in alkaline seawater. Especially, there was no evident decay in overpotential, indicating the excellent stability of NiTe@NiFe-LDH/NF. After 50-h stability test, the morphology of NiTe@NiFe-LDH/NF in Figure S7 demonstrated the preservation of the core-shell structure. Additionally, the XRD pattern of NiTe@NiFe-LDH/NF (Figure S8) displayed that no additional peaks were observed compared with the initial catalyst. The XPS spectrum (Figure S9) in the Ni 2P region revealed two additional peaks at 857.7 and 874.5 eV, corresponding to Ni^3+^ in NiOOH. It was demonstrated that NiOOH, an active phase for OER, was formed during the OER process.^24^^,^^52^^,^^53^^,^^54^ The XPS spectra of the O 1s region unveiled an additional peak at 530.0 eV for metal-O (referred to as O3).^55^^,^^56^

### Conclusions

In summary, we have successfully fabricated a core-shell NiTe@NiFe-LDH electrocatalyst customized for seawater oxidation. To drive a *j* of 100 mA cm^−2^ in alkaline seawater, NiTe@NiFe-LDH/NF only necessitates a low overpotential of 277 mV. Compared to NiFe-LDH directly grown on NF, the incorporated NiTe nanorod core provides an elevated electrochemical surface area, thereby facilitating an increased number of active sites. Furthermore, the real active species NiOOH for alkaline seawater oxidation are generated through reconstruction. Importantly, it exhibited robust stability during an extended 50-h assessment. This work not only advances the field by presenting a proficient OER electrocatalyst but also broadens the potential for future exploration into hierarchical nanostructures tailored for seawater oxidation applications.

### Limitations of the study

Our work has demonstrated an excellent electrocatalyst for seawater oxidation with low overpotential and robust stability. Based on the combination of EDS and XPS, increased active surface area and improved conductivity have been interpreted as the key factors that facilitate OER performance. However, an in-depth understanding of the catalytic process and an analysis of the oxidation states of the catalyst remain significant. Hence, we will make a further exploration by *in situ* characterizations and theoretical calculations.

## STAR★Methods

### Key resources table

**Table:** 

REAGENT or RESOURCE	SOURCE	IDENTIFIER
**Chemicals, peptides, and recombinant proteins**		

Ni(NO_3_)_2_·6H_2_O	Chengdu Kelong Chemical Reagent Factory	13478-00-7
NaCl	Chengdu Kelong Chemical Reagent Factory	7647-14-5
KOH	Chengdu Kelong Chemical Reagent Factory	1310-58-3
C_2_H_5_OH	Chengdu Kelong Chemical Reagent Factory	64-17-5
HCl	Chengdu Kelong Chemical Reagent Factory	7647-01-0
Na_2_CO_3_	Chengdu Kelong Chemical Reagent Factory	497-19-8
FeSO_4_·7H_2_O	Shanghai Macklin Biochemical Co., Ltd.	7782-63-0
Na_2_TeO_3_	Shanghai Macklin Biochemical Co., Ltd.	10102-20-2
N_2_H_4_·H_2_O	Chengdu Jinshan Chemical Reagent Co., Ltd.	7803-57-8
Ni foam	Shenzhen Green and Creative Environmental Science and Technology Co., Ltd.	/

### Resource availability

#### Lead contact

Further information and requests for resources should be directed to and will be fulfilled by the lead contact, Dr. Xuping Sun (xpsun@uestc.edu.cn).

#### Materials availability

This study did not generate new unique reagents. All chemicals were obtained from commercial resources and used as received.

#### Data and code availability

• Data reported in this paper will be shared by the lead contacts upon reasonable request.
• This study does not report any original code.
• Any additional information required to reanalyze the data reported in this paper is available from the lead contacts upon reasonable request.

### Experimental model and study participant details

For all the experiments, both catalyst synthesis and electrochemical tests were carried out at ambient conditions at University of Electronic Science and Technology of China. All the experiments involve no laboratory animals.

### Method details

#### Synthesis of NiTe/NF

0.886 g Na_2_TeO_3_ was first dissolved in 40 mL deionized water. Then, 24 mL N_2_H_4_·H_2_O was dropwise slowly added to the above solution with continuous stirring. Meanwhile, NF cut into 2 cm × 4 cm were sonicated in 3 M HCl, C_2_H_5_OH, and deionized water, respectively for 10 min before being put into a 100 mL Teflon lined autoclave along with the abovementioned precursor solution. After heating at 180°C for 24 h, the NF with NiTe nanoarrays was cleaned with distilled water and ethanol, respectively. The as-prepared NiTe nanoarrays were dried under 70°C for 6 h for the next step electrodeposition.

#### Synthesis of NiTe@NiFe-LDH/NF

NiFe-LDH was deposited on the NiTe/NF surface by a simple electrodeposition technique. Briefly, 2.085 g Fe(SO_4_)⋅7H_2_O and 2.181 g Ni(NO_3_)_2_⋅6H_2_O were dissolved in 50 mL deionized water and a typical three-electrode system was employed in the electrodeposition process, in which NiTe/NF, Pt plate and Ag/AgCl were used as working electrode, counter electrode and reference electrode, respectively. The solution was purged with nitrogen gas for 0.5 h before the experiment. The electrodeposition was carried out at a potential of −1.0 V for 60 s. Various electrodeposition times ranging from 50 s to 80 s were investigated to determine the optimal performance. Catalyst loading was obtained from mass increased before and after the synthesis process.

#### Characterizations

X-ray diffraction (XRD) data was acquired from a LabX XRD-6100 X-ray diffractometer with a Cu Kα radiation (40 kV, 30 mA) of wavelength of 0.154 nm (SHIMADZU, Japan). Scanning electron microscope (SEM) images were collected on a GeminiSEM 300 scanning electron microscope (ZEISS, Germany) at an accelerating voltage of 5 kV. Transmission electron microscopy (TEM), high-angle annular dark-field scanning TEM (HAADF-STEM), and Energy dispersive spectrometer (EDS) images were acquired on a JEM-2800 electron microscope (JEOL, Japan) operated at 200 kV. X-ray photoelectron spectroscopy (XPS) measurements were performed on an ESCALABMKII X-ray photoelectron spectrometer using Mg as the exciting source.

#### Electrochemical measurements

Electrochemical OER experiments were performed with the CHI 760E electrochemical workstation, using the prepared samples (1 × 0.5 cm^2^), carbon rod, and Hg/HgO as the working electrode, counter electrode, and reference electrode, respectively. All the potentials in this experiment are presented as reversible hydrogen electrodes (RHE): E (vs. RHE) = E (vs. Hg/HgO) + 0.0591 × pH + 0.098. Electrochemical impedance spectroscopy (EIS) measurements were performed at 1.52 V vs. RHE from 10^5^ to 0.01 Hz with an amplitude of 5 mV. The iR-compensated potential was obtained after the correction of solution resistance measured following the equation: E_corr_ = E − iR, where E is the original potential, R the solution resistance, i the corresponding current, and E_corr_ the iR-compensated potential.

### Quantification and statistical analysis

We did not perform any statistical analysis to filter out relevant data.
