# Supplementary material for: Fabrication of a hierarchical NiTe@NiFe-LDH core-shell array for high-efficiency alkaline seawater oxidation
Source: iScience. 2023 Dec 15;27(1):108736. doi: 10.1016/j.isci.2023.108736 (PMC10805641; doi:10.1016/j.isci.2023.108736)
Supplement: Document S1. Figures S1–S9 and Tables S1 [file mmc1.pdf]

iScience, Volume *27*

Supplemental information

Fabrication of a hierarchical NiTe@NiFe-LDH core-shell array for high-efficiency alkaline seawater oxidation

Xuexuan Ju, Xun He, Yuntong Sun, Zhengwei Cai, Shengjun Sun, Yongchao Yao, Zixiao Li, Jun Li, Yan Wang, Yuchun Ren, Binwu Ying, Yongsong Luo, Dongdong Zheng, Qian Liu, Lisi Xie, Tingshuai Li, Xuping Sun, and Bo Tang

Supplementary Information

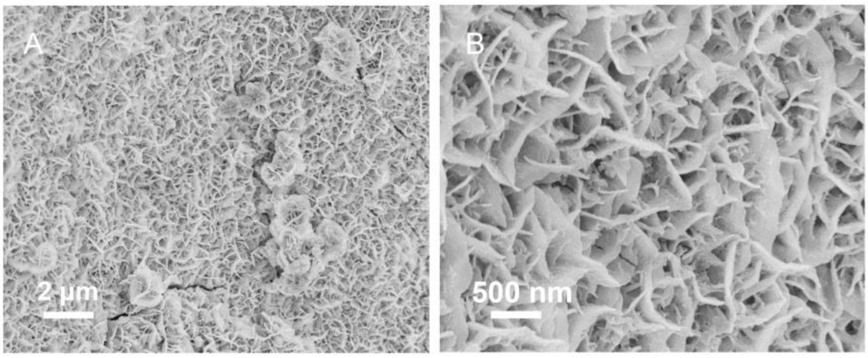

Figure. S1. (A) Low- and (B) high-magnification SEM images of NiFe-LDH/NF, related to Figure 1.

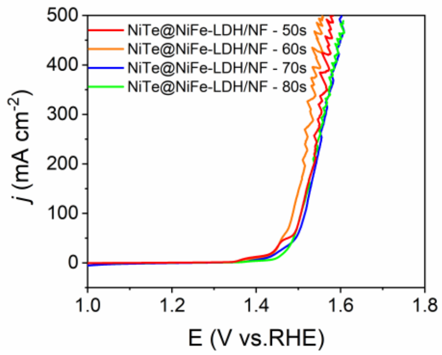

Figure. S2. LSV curves of NiTe@NiFe-LDH/NF at different electrodeposition times in 1 M KOH, related to Figure 3.

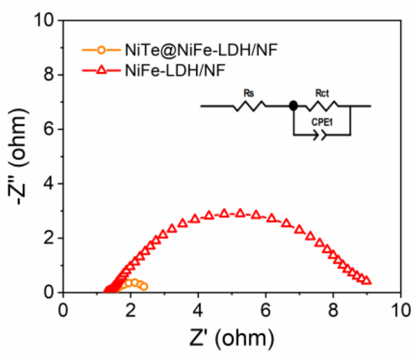

Figure. S3. Nyquist plots of NiTe@NiFe-LDH/NF and NiFe-LDH/NF in 1 M KOH, related to Figure 3.

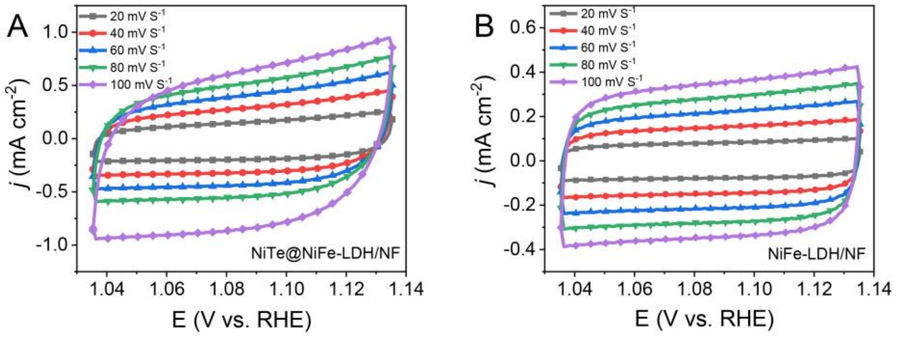

Figure. S4. CV curves of (A) NiTe@NiFe-LDH/NF and (B) NiFe-LDH/NF from 1.035 V to 1.135 V in 1 M KOH, related to Figure 3.

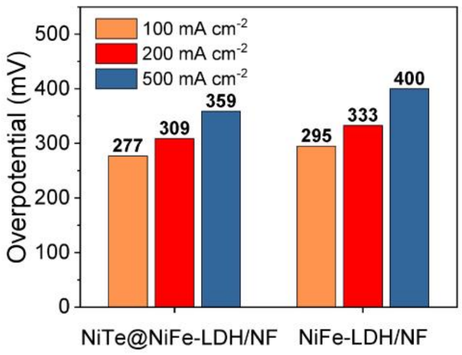

Figure S5. Corresponding overpotentials of the NiTe@NiFe-LDH/NF and NiFe-LDH/NF in alkaline seawater, related to Figure 4.

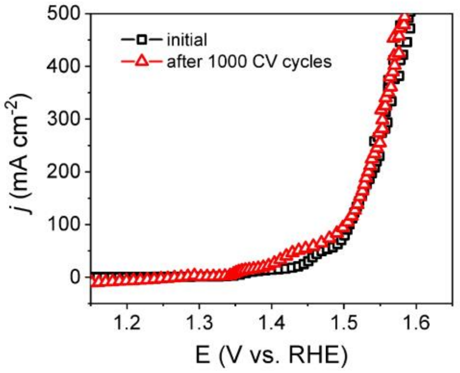

Figure S6. LSV curves of NiTe@NiFe-LDH/NF before and after 1000 CV cycles, related to Figure 4.

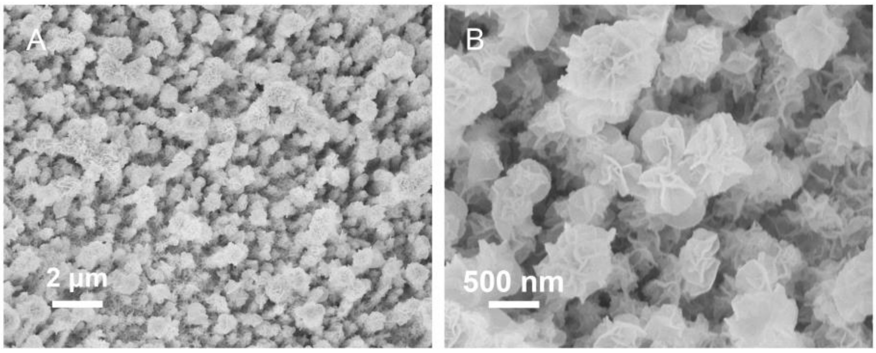

Figure S7. (A) Low- and (B) high-magnification SEM images of NiTe@NiFe-LDH/NF after stability test in alkaline seawater, related to Figure 4.

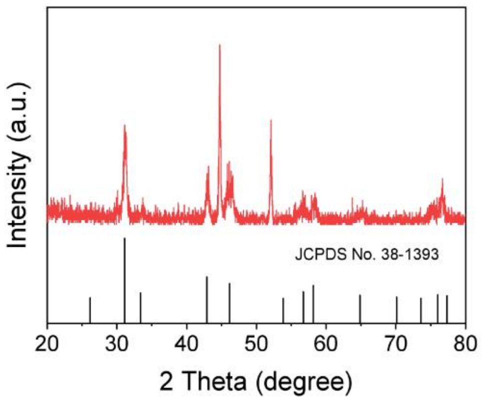

Figure S8. XRD pattern of NiTe@NiFe-LDH/NF after stability test in alkaline seawater, related to Figure 4.

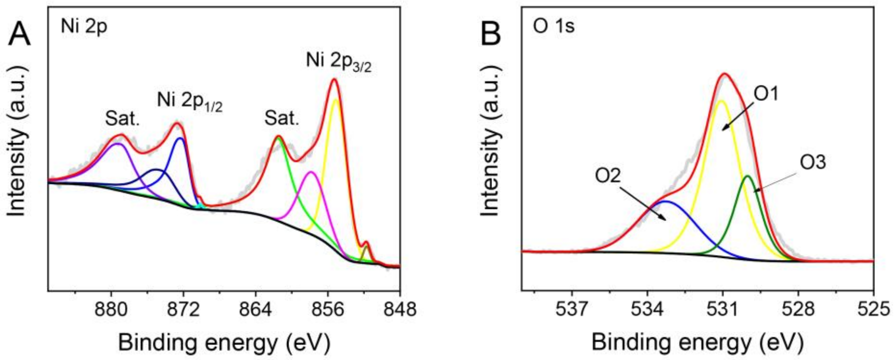

Figure S9. XPS spectra of NiTe@NiFe-LDH in the (A) Ni 2p and (B) O 1s regions after stability test in alkaline seawater, related to Figure 4.

Table S1. Comparisons of the OER performance of NiTe@NiFe-LDH/NF and other electrocatalysts in alkaline seawater, related to Figure 4.

Catalyst (1 M KOH + seawater)	Current density ($mA\ cm^{-2}$)	Corresponding overpotential (mV)	Tafel slope ($mV\ dec^{-1}$)	Catalyst loading ($mg\ cm^{-2}$)	Ref.
NiTe@NiFe LDH/NF	100	277	68.66	3.00	This work
MnO_x/NiFe LDH/NF	100	276	77.00	/	[1]
N-CDs/NiFe LDH/NF	100	340	43.40	2.60	[2]
NiFe LDH/CC	100	301	26.00	/	[3]
NiFe LDH@$FeNi_2S_4$/NF	100	271	29.40	3.60	[4]
NiFe LDH-S/CC	100	296	233.00	0.28	[5]
BZ-NiFe LDH/CC	100	300	45.06	1.60	[6]
Gd-Mn_3O_4@ CuO-$Cu(OH)_2$	100	350	63.00	/	[7]
Fe-Co-S/Cu_2O/Cu	100	440	111.00	/	[8]
NiMoN@NiFeN/NF	100	307	58.60	1.27	[9]
Ni_2P-Fe_2P/NF	100	305	58.00	/	[10]
S-(Ni,Fe)OOH/NF	100	300	48.90	/	[11]
S-$NiMoO_4$@NiFe-LDH/NF	100	315	90.00	/	[12]
$Ni(OH)_2$-TCNQ/GP	100	340	75.00	/	[13]
Fe-CoCH/NF	100	317	45.00	1.50	[14]

References

[1] Wang, Z., Wang, C., Ye, L., Liu, X., Xin, L., Yang, Y., Wang, L., Hou, W., Wen, Y., and Zhan, T. (2022). MnO_x film-coated NiFe-LDH nanosheets on Ni foam as selective oxygen evolution electrocatalysts for alkaline seawater oxidation. Inorg. Chem. *61*, 15256–15265. https://doi.org/10.1021/acs.inorgchem.2c02579.

[2] Ding, P., Song, H., Chang, J., and Lu, S. (2022). N-doped carbon dots coupled NiFe-LDH hybrids for robust electrocatalytic alkaline water and seawater oxidation. Nano Res. *15*, 7063–7070. https://doi.org/10.1007/s12274-022-4377-4.

[3] Dong, G., Xie, F., Kou, F., Chen, T., Wang, F., Zhou, Y., Wu, K., Du, S., Fang, M., and Ho, J.C. (2021). NiFe-layered double hydroxide arrays for oxygen evolution reaction in fresh water and seawater. Mater. Today Energy *22*, 100883. https://doi.org/10.1016/j.mtener.2021.100883.

[4] Tan, L., Yu, J., Wang, C., Wang, H., Liu, X., Gao, H., Xin, L., Liu, D., Hou, W., and Zhan, T. (2022). Partial sulfidation strategy to NiFe-LDH@$FeNi_2S_4$ heterostructure enable high-performance water/seawater oxidation. Adv. Funct. Mater. *32*, 2200951. https://doi.org/10.1002/adfm.202200951.

[5] Jung, S.Y., Kang, S., Kim, K.M., Mhin, S., Kim, J.C., Kim, S.J., Enkhtuvshin, E., Choi, S., and Han, H. (2021). Sulfur-incorporated nickel-iron layered double hydroxides for effective oxygen evolution reaction in seawater. Appl. Surf. Sci. *568*, 150965. https://doi.org/10.1016/j.apsusc.2021.150965.

[6] Zhang, L., Liang, J., Yue, L., Dong, K., Li, J., Zhao, D., Li, Z., Sun, S., Luo, Y., Liu, Q., et al. (2022). Benzoate anions-intercalated NiFe-layered double hydroxide nanosheet array with enhanced stability for electrochemical seawater oxidation. Nano Res. Energy *1*, e9120028. https://doi.org/10.26599/NRE.2022.9120028.

[7] ul Haq, T., Mansour, S., and Haik, Y. (2022). Electronic and structural modification of Mn_3O_4 nanosheets for selective and sustained seawater oxidation. ACS Appl. Mater. Interface *14*, 20443–20454. https://doi.org/10.1021/acsami.1c24304.

[8] Sun, J., Song, P., Zhou, H., Lang, L., Shen, X., Liu, Y., Cheng, X., Fu, X., and Zhu, G. (2021). A surface configuration strategy to hierarchical Fe-Co-S/Cu_2O/Cu electrodes for oxygen evolution in water/seawater splitting. Appl. Surf. Sci. *567*, 150757. https://doi.org/10.1016/j.apsusc.2021.150757.

[9] Yu, L., Zhu, Q., Song, S., McElhenny, B., Wang, D., Wu, C., Qin, Z., Bao, J., Yu, Y., Chen, S., et al. (2019). Non-noble metal-nitride based electrocatalysts for high-performance alkaline seawater electrolysis. Nat. Commun. *10*, 5106. https://doi.org/10.1038/s41467-019-13092-7.

[10] Wu, L., Yu, L., Zhang, F., McElhenny, B., Luo, D., Karim, A., Chen, S., and Ren, Z. (2021). Heterogeneous bimetallic phosphide Ni_2P-Fe_2P as an efficient bifunctional catalyst for water/seawater Splitting. Adv. Funct. Mater. *31*, 2006484. https://doi.org/10.1002/adfm.202006484.

[11] Yu, L., Wu, L., McElhenny, B., Song, S., Luo, D., Zhang, F., Yu, Y., Chen, S., and Ren, Z. (2020). Ultrafast room-temperature synthesis of porous S-doped Ni/Fe (oxy)hydroxide electrodes for oxygen evolution catalysis in seawater splitting. Energy Environ. Sci. *13*, 3439–3446. https://doi.org/10.1039/D0EE00921K.

[12] Wang, H., Chen, L., Tan, L., Liu, X., Wen, Y., Hou, W., and Zhan, T. (2022). Electrodeposition of NiFe-layered double hydroxide layer on sulfur-modified nickel molybdate nanorods for highly efficient seawater splitting. J. Colloid Interface Sci. *613*, 349–358. https://doi.org/10.1016/j.jcis.2022.01.044.

[13] Zhang, L., Wang, J., Liu, P., Liang, J., Luo, Y., Cui, G., Tang, B., Liu, Q., Yan, X., Hao, H., et al. (2022). $Ni(OH)_2$ nanoparticles encapsulated in conductive nanowire array for high-performance alkaline seawater oxidation. Nano Res. *15*, 6084–6090. https://doi.org/10.1007/s12274-022-4391-6.

[14] Shi, S., Sun, S., He, X., Zhang, L., Zhang, H., Dong, K., Cai, Z., Zheng, D., Sun, Y., Luo, Y., et al. (2023). Improved electrochemical alkaline seawater oxidation over cobalt carbonate hydroxide nanowire array by iron doping. Inorg Chem. *62*, 11746–11750. https://doi.org/10.1021/acs.inorgchem.3c01473.
